# LIMPRINT in Australia

**DOI:** 10.1089/lrb.2018.0087

**Published:** 2019-04-17

**Authors:** Susan J. Gordon, Susie G. Murray, Trudie Sutton, Marie-Michelle Coulombe, Sally J. James, Malou Van Zanten, Joanne K. Lawson, Christine Moffatt

**Affiliations:** ^1^ College of Nursing and Health Sciences, Flinders University, Adelaide, Australia.; ^2^ Centre for Research and Implementation of Clinical Practice, London, United Kingdom.; ^3^ General Manager Aged Care Housing Group, Mile End, South Australia, Australia.; ^4^ Calvary Public Hospital, Australian Capital Territory, Australia.; ^5^ Western Health Chronic Wound Service, St Albans, Australia.; ^6^ Flinders Centre for Innovation in Cancer, Flinders University, Adelaide, Australia.; ^7^ School of Social Sciences, Nottingham University, Nottingham, United Kingdom.

**Keywords:** edema, health services, wounds, lymphedema, lymphoedema

## Abstract

***Background and Study Objective:*** Australia was one of nine participating countries in the epidemiology Phase II Lymphoedema Impact and Prevalence – International (LIMPRINT) project to determine the number of people with chronic edema (CO) in local health services.

***Methods and Results:*** Data collection occurred through questionnaire-based interviews and clinical assessment with provided LIMPRINT tools. Four different types of services across three states in Australia participated. A total of 222 adults participated with an age range from 22 to 102 years, and 60% were female. Site 1 included three residential care facilities (54% of participants had swelling), site 2 was community-delivered aged care services (24% of participants had swelling), site 3 was a hospital setting (facility-based prevalence study; 28% of participants had swelling), and site 4 was a wound treatment center (specific patient population; 100% of participants had swelling). Of those with CO or secondary lymphedema, 93% were not related to cancer, the lower limbs were affected in 51% of cases, and 18% of participants with swelling reported one or more episodes of cellulitis in the previous year. Wounds were identified in 47% (*n* = 105) of all participants with more than half of those with wounds coming from the dedicated wound clinic. Leg/foot ulcer was the most common type of wound (65%, *n* = 68).

***Conclusions:*** Distances between services, lack of specialized services, and various state funding models contribute to inequities in CO treatment. Understanding the high number of noncancer-related CO presentations will assist health services to provide timely effective care and improve referral pathways.

## Background and Methods

Four different types of services participated in the Australian Lymphoedeme Impact and Prevalence – International (LIMPRINT) project.

### Description of services

Aged Care Housing Pty Ltd (ACH) is a not-for-profit provider of residential aged care and community services delivered in peoples' homes in Adelaide, South Australia. They do not provide specific lymphedema services but provide general care of swelling and wounds as needed in both settings. Funding is not specific to either the management of swelling or wound care but is bundled into the Australian funding packages for aged care services.

Service 1 included three ACH group residential care facilities dedicated to creating opportunities for older people to thrive, where they are valued, stimulated, and connected, while still retaining control of their lives. High-quality care is provided by vocationally trained aged care workers supported by registered nurses, General Practitioners, and a range of health professionals to optimize lifestyle, social, and accommodation choices based on individual interests.

Service 2 was the ACH group community-based health and wellness services which provide a range of allied health, nursing, and complimentary services to community-based clients. Clients are primarily older than 65 years and have a level of government funding to support their access and provision of services.

Data collection occurred in January and February 2017. Clients from both services were recruited based on a known history of chronic edema (CO) with or without a diagnosis of lymphedema. Recruitment was on a voluntary basis. The data were collected by health professionals, including nurses, physiotherapists, exercise scientists, and personal trainers. The current funding models for wound and swelling management for the services are provided primarily by government-supported funding models, including Home Care Packages, the Commonwealth Home Support Program (CHSP), or as part of normal or routine care for resident within a residential care facility.

Calvary Public Hospital Bruce (CPHB) (Service 3) is an acute and subacute hospital facility serving the northern suburbs of Canberra, Australian Capital Territory (ACT). CPHB ambulatory services include an outpatient specialist lymphedema service. This service runs out of the outpatient physiotherapy department and is the hub in the ACT for specialist management of lymphedema. The CPHB Lymphedema Service provides an outpatient service for patients for the whole of the ACT and surrounding regions. The CPHB lymphedema therapists also provide support and consultation to nursing, medical, and allied health professionals on the inpatient wards, but there is limited capacity for comprehensive lymphedema or CO management. This predominantly outpatient service is government funded. Compression garments to assist in the management of lymphedema or CO can be obtained through government-funded bodies to eligible patients (usually those receiving an Australian pension).

Six lymphedema physiotherapists at the CPHB performed a half-day audit on the Aged Care and Rehabilitation ward on October 21, 2016, and a full-day audit of other inpatient wards (including the Coronary Care Unit, Short Stay Unit, the Medical Assessment and Planning Unit, surgical and medical wards) on October 26, 2016. All inpatients present on the wards at the time of the audit were approached. The study was described to patients and consent was sought. If consent was gained, a questionnaire-based interview and clinical assessment using the LIMPRINT assessment tools^1^ was performed. Inpatients excluded from the study were patients younger than 18 years, patients receiving end-of-life care, and patients unwilling or unable to participate. Of the 142 patients who were approached on the wards, 113 patients participated in the study.

The Western Health Chronic Wound Service (WH CWS) (Service 4) is a specialist wound treatment center located in Melbourne, Victoria, within an acute hospital outpatient setting. All clients attending the WH CWS have wounds that are older than 4 weeks in duration. Chronic leg edema and lymphedema on the same leg as the wound is frequently found in those attending the clinic. This population group lives within the geographical western suburbs of Melbourne, comprising a diverse cultural mix, speaking some 100 languages and dialects. Attendance at the clinic involves no charge to patients, as it is a Medicare-funded service. However, when the patients are attending to their wound dressings outside the clinic, they mostly purchase their own dressing supplies and compression therapy, as no funding for these is available in Victoria. All patients attending the clinic during a 10-week-period were screened to participate in the LIMPRINT study by the Clinical Nurse Consultant—Wound/Lymphedema who saw them during their normal scheduled appointment. Fifty-five of 57 patients attending the clinic had chronic leg edema and agreed to participate in the study.

People who attended Services 1 and 2 were not seeking specific care for wounds or swelling but needed assistance generally with functional activities or conditions. Those attending Service 3 were people seeking acute or subacute care not specific to wounds or swelling, whereas those attending Service 4 had known wounds, which required attention.

## Overall Findings

Across the four services, 222 participants were recruited to participate in the study. The majority of participants were female (60%, *n* = 132) and ranged in age from 22 to 102 years (Table 1). While many of the participants were of normal weight (41%, *n* = 89), more than 50% were obese (34%, *n* = 74) or morbidly obese (17%, *n* = 37).

**Table 1.** Age, Gender, and Weight of Participants at Each Service

*Participants*	*Service 1 (*N* = 37) (100%)*		*Service 2 (*N* = 17) (100%)*		*Service 3 (*N* = 113) (100%)*		*Service 4 (*N* = 55) (100%)*		*Total (*N*)*
Male	*n* = 5	14%	*n* = 4	24%	*n* = 49	43%	*n* = 32	58%	*N* = 222
Female	*n* = 32	86%	*n* = 13	76%	*n* = 64	57%	*n* = 23	42%	
Median age (range) male	91 (71–102)		74 (68–86)		74 (36–90)		64 (21–91)		
Median age (range) female	90 (67–92)		76 (69–90)		74 (22–99)		70 (30–89)		
Normal weight									
Male	2	5%	4	24%	16	14%	8	15%	*N* = 89 (40%)
Female	19	51%	9	53%	28	25%	3	5%	
Under weight									
Male	0	0	0	0	5	4.4%	2	4%	*N* = 22 (10%)
Female	4	11%	0	0	8	7.1%	3	5%	
Obese									
Male	3	8%	0	0	23	20%	10	19%	*N* = 74 (34%)
Female	9	24%	4	24%	20	18%	5	9%	
Morbidly obese									
Male	0	0	0	0	8	7.1%	12	21.8%	*N* = 37 (17%)
Female	0	0	0	0	5	4.4%	12	21.8%	

Primary lymphedema was reported in 7% of all participants (*n* = 15) and secondary lymphedema/CO in 43% (*n* = 95) of participants. Further reporting in this article regarding secondary lymphedema/CO will be denoted as CO, which encompasses all participants with edema for a minimum of 3 months irrespective of the cause. Of the participants who had CO, 6% (*n* = 6) was related to cancer treatment, 1% (*n* = 1) to metastatic lymphatic obstruction, and 93% (*n* = 87) to noncancer-related causes. Where swelling was noncancer-related, venous problems were present in 80% (*n* = 70) of participants, and both immobility and obesity were reported to contribute to swelling in ∼39% (*n* = 34, immobility; *n* = 33, obesity) of participants with CO.

In the facilities that were not dedicated to the management of wounds (Services 1, 2, and 3), 1% (*n* = 2) had primary lymphedema and 35% (*n* = 53) had CO. The majority were from the residential aged care facility where more than 50% (*n* = 21) had CO, of which 82% (*n* = 18) were venous related.

Cellulitis had occurred in the area affected by swelling in 37% (*n* = 41) of participants, and 15% (*n* = 16) had been hospitalized in the past year due to cellulitis (Table 2). Most often, people were admitted to hospital with cellulitis on one (69%, *n* = 11) or two occasions (25%, *n* = 4) during the past year. Wounds were identified in 47% (*n* = 105) of all participants, with leg/foot ulcer the most common type of wound (65%, *n* = 68), then surgical wounds (17%, *n* = 18) and other types of wounds (18%, *n* = 19) (Table 2).

**Table 2.** Type of Edema, Cellulitis, and Wounds Per Service

	*Service 1 (*N* = 37)*		*Service 2 (*N* = 17)*		*Service 3 (*N* = 113)*		*Service 4 (*N* = 55)*		*Total* n *(%)*
	n	*%*	n	*%*	n	*%*	n	*%*	
Primary lymphedema	1	3	0	0	1	1	13	24	16 (7)
CO/secondary lymphedema	19	51	4	24	30	27	42	76	94 (42)
Total %		54		34		28		100	
Cancer related	2	5	0	0	3	3	1	1	6 (6)
Cellulitis	1	3	1	6	12	11	27	49	41 (18)
Wounds	11	30	1	6	38	34	55	100	105 (47)

CO, chronic edema.

The most common site of swelling were the lower limbs (51%, *n* = 112), followed by the upper limbs (2%, *n* = 5) and midline (2%, *n* = 4). Of the 110 participants who had primary lymphedema or CO, 41% (*n* = 45) reported their swelling was well controlled.

A number of comorbidities were reported, including diabetes mellitus (20%, *n* = 44), heart failure or ischemic heart disease (20%, *n* = 43), neurological disorder (15%, *n* = 34), and peripheral arterial disease (16%, *n* = 36) (Table 3).

**Table 3.** Relevant Comorbidities for All Participants at Each Service

*Relevant comorbidities*	*Service 1 (*N* = 37)*		*Service 2 (*N* = 17)*		*Service 3 (*N * = 113)*		*Service 4 (*N* = 55)*		*Total*
	n	*%*	n	*%*	n	*%*	n	*%*	
Diabetes mellitus	4	11	0	0	23	20	17	31	44
Heart failure	8	22	0	0	30	27	5	9	43
Neurological disorder	7	19	1	6	25	22	1	2	34
Peripheral arterial disease	6	16	2	12	10	9	18	33	36

The majority of participants reported that they have had swelling for more than 10 years (31%, *n* = 34), with less than 6 months and 2–5 years each accounting for a further 18% of participants (Table 4).

**Table 4.** Duration of Edema for Participants at Each Service

	*Service 1 (CO* n* = 20)*	*Service 2 (CO* n* = 4)*	*Service 3 (CO* n* = 31)*	*Service 4 (CO* n* = 55)*	*Total (CO* n* = 110)*
<6 months	5		7	8	20
6–12 months	2		5	2	9
1–2 years	5		5	4	14
2–5 years	2		8	9	19
5–10 years	1	2	2	9	14
>10 years	5	2	4	23	34
Total	20	4	31	55	110

## Service-Specific Information

### Residential aged care facility (Adelaide)

Of the 37 participants at Service 1, 1 participant had primary lymphedema and 19 had CO. Of the 19 with CO, 2 were related to cancer and 17 noncancer, of which the majority (*n* = 15) were venous related. Twenty-five cases (68%) had comorbidities with the majority suffering from heart failure (*n* = 8, 22%). Eleven (30%) participants had wounds, 73% (*n* = 8) in the leg/foot and 17% (*n* = 3) in the hand/arm. Participants mostly had swelling for less than 6 months (*n* = 5), 1–2 years (*n* = 5), or more than 10 years (*n* = 5). Of the total 20 participants with swelling, 15 reported that the swelling was under control.

In this setting, the cost and access to services for swelling and wounds is controlled and supplied for by the aged care facility. Although edema was not the main reason for remaining in the care facility, it is unclear if there was adequate provision in the funding models to support best practice management of swelling and wounds.

### Community aged care services (Adelaide)

Of the 17 participants at this service, 4 participants had CO, which were reported as noncancer-related venous swelling. Only three participants had comorbidities, two with peripheral arterial disease and one with a neurological disorder. One participant was reported to have a wound (6%), which was a leg/foot ulcer. Of the four participants with swelling, 50% (*n* = 2) had swelling present for 5–10 years and 50% (*n* = 2) for more than 10 years. Two reported that the swelling was under control (50%), and two reported that it was not (50%).

This setting is focused on providing community-based aged care services. Most participants receive services for a considerable amount of time, and their care is not related to swelling or wounds. Of those with swelling, one participant reported free treatment that was covered by health care insurance, whereas four had no insurance cover at all. Two participants were able to pay for treatment, and four had treatment within a reasonable travel distance.

### Hospital inpatient, Calvary Public Hospital (Canberra)

Of the 113 participants at this service, 28% (*n* = 31) had swelling, of which 1 was primary lymphedema and 30 were CO. CO was subdivided into two categories: 2 participants had cancer-related CO and 28 noncancer-related CO. Of the 28 participants with noncancer-related CO, 14 were deemed to be of venous etiology. Overall, 78 comorbidities were found; 27% (*n* = 30) had heart failure/ischemic heart disease; 22% (*n* = 25) had neurological disorders; 20% (*n* = 23) had diabetes mellitus; and 9% (*n* = 10) had peripheral arterial disease. A total of 38 wounds were reported (*n* = 38, 34%), with the leg the most common site (*n* = 20, 59%). The majority were classified as surgical wounds (*n* = 19, 56%). Most participants had swelling from 2 to 5 years (*n* = 8, 26%) or less than 6 months (*n* = 7, 23%). Of the total of 31 participants with swelling, 29% (*n* = 9) reported that that it was under control.

Of the 29 participants with swelling to their lower limbs, 38% (*n* = 11) reported a history of lower limb cellulitis.

In this hospital setting, three participants with swelling reported that they did not have access to free treatment within their health care system and two reported that they could not afford to pay for care. Two participants reported that treatment was not available within a reasonable travel distance and five were unable to access service due to the distance or lack of transport. At the CPHB site, six participants' swelling or wound prevented their discharge from the hospital facility.

### Western Health Chronic Wound Service (Melbourne)

All participants at this service had swelling. Primary lymphedema was classified in 13 cases (24%) and CO in 42 cases (76%), of which 41 were noncancer-related swelling. The noncancer-related swelling was subdivided into 38 participants (69%) with venous-related swelling, 19 participants (35%) with immobility-related swelling, and in 25 participants (45%), obesity was considered to contribute to the CO. The most reported comorbidities were identified as peripheral arterial disease (33%, *n* = 18), followed by diabetes mellitus (31%, *n* = 17), heart failure (9%, *n* = 5), and neurological disorder (2%, *n* = 1). The duration of the swelling was most commonly present for more than 10 years (42%, *n* = 23) (Table 4). As this was a specialist wound care treatment service, 100% of participants had one or more wounds. Most commonly, the leg was affected by swelling (95%, *n* = 52), and leg/foot ulcer was present in 95% of all cases (*n* = 52). Overall, 19 (35%) participants self-reported that their swelling was under control, with 36 participants (65%) reporting that their swelling was not under control. Forty-nine percent (*n* = 27) of participants reported a history of cellulitis in the area affected by swelling.

In the wound clinic setting, only two participants reported access to free treatment, both being work cover recipients. The majority (96%) of participants reported no access to free treatment. Three participants reported that they were unable to pay for care. Although only one participant reported that there were no services available in a reasonable travel distance, five participants were deterred from accessing services due to distance and availability of public transport.

## Conclusions

Across the three services that were not dedicated to wound care, between 28% and 54% of participants had primary lymphedema or CO. The highest rate was in the residential aged care facility where the age was higher than at other sites, and it is likely that the general level of mobility was less. As well high levels of comorbidities were reported and are likely to have contributed to these presentations. Importantly, all participants presenting to the wound clinic (Service 4) had swelling.

This study demonstrated that within an Australian hospital setting, the proportion of patients with CO are predominantly of a noncancer-related cause. Currently, the referral pathways for patients at risk of or presenting with CO related to cancer treatment are well defined and understood by medical professionals. This facilitates early detection and intervention for this patient cohort, whereas the noncancer-related patients are often referred at a much later stage or not at all. An increased understanding of this cohort of patients will help us provide more timely and effective care and address better referral pathways.

Australia is a large country, and public transport systems are often difficult to access. The outpatient lymphedema service at CPHB (Service 3) covers all of the ACT as well as regional New South Wales. Distance has a significant impact on accessing care. ACT Health has identified a need to shift the focus of services from the hospital to the community environment and have since introduced a “hub and spoke” model where complex CO and lymphedema management is performed in the hub and noncomplex management occurs in community health centers. Even the Melbourne metropolitan-based wound clinic (Service 4) covers a geographical area of some 1569 km^2^.

The funding models for the provision of services of this type vary between states. Even within states, the provision of services between health regions may vary, including different services, which may be publicly funded by government or privately funded. For instance, Service 4 is an entirely Victorian public health service. Health insurance makes no difference and does not cover costs for dressing supplies or compression therapy. Service 3 (CPHB) is a public health service; therefore, patients do not pay for care but have to purchase their own compression garments. Compression garments may be partially or fully funded through government-funded schemes for eligible patients. Occasionally, patients are be able to use their private health insurance to help pay for compression garments. Meanwhile, costs related to the management of swelling and wounds in aged care services are paid from a bundled fee that has no specific allocation.

The high rates of cellulitis at Service 4 (a dedicated wound clinic) might reflect that cellulitis is both a risk of and a risk for CO. Certainly, there would be benefit from lymphedema-related assessment and management for this group.

## References

[1] Moffatt CJ, Susie M, Mercier G, Quere I, Franks P. The development and validation of the LIMPRINT methodology. Lymphat Res Biol. (Suppl) 10.1089/lrb.2018.0081 PMC6636670 30995185

